# Environmental rhythms orchestrate neural activity at multiple stages of processing during memory encoding: Evidence from event-related potentials

**DOI:** 10.1371/journal.pone.0234668

**Published:** 2020-11-18

**Authors:** Paige Hickey, Annie Barnett-Young, Aniruddh D. Patel, Elizabeth Race

**Affiliations:** 1 Department of Psychology, Tufts University, Medford, Massachusetts, United States of America; 2 Program in Brain, Mind, and Consciousness, Canadian Institute for Advanced Research (CIFAR), Toronto, Ontario, Canada; Harvard Medical School, UNITED STATES

## Abstract

Accumulating evidence suggests that rhythmic temporal structures in the environment influence memory formation. For example, stimuli that appear in synchrony with the beat of background, environmental rhythms are better remembered than stimuli that appear out-of-synchrony with the beat. This rhythmic modulation of memory has been linked to entrained neural oscillations which are proposed to act as a mechanism of selective attention that prioritize processing of events that coincide with the beat. However, it is currently unclear whether rhythm influences memory formation by influencing early (sensory) or late (post-perceptual) processing of stimuli. The current study used stimulus-locked event-related potentials (ERPs) to investigate the locus of stimulus processing at which rhythm temporal cues operate in the service of memory formation. Participants viewed a series of visual objects that either appeared in-synchrony or out-of-synchrony with the beat of background music and made a semantic classification (living/non-living) for each object. Participants’ memory for the objects was then tested (in silence). The timing of stimulus presentation during encoding (in-synchrony or out-of-synchrony with the background beat) influenced later ERPs associated with post-perceptual selection and orienting attention in time rather than earlier ERPs associated with sensory processing. The magnitude of post-perceptual ERPs also differed according to whether or not participants demonstrated a mnemonic benefit for in-synchrony compared to out-of-synchrony stimuli, and was related to the magnitude of the rhythmic modulation of memory performance across participants. These results support two prominent theories in the field, the Dynamic Attending Theory and the Oscillation Selection Hypothesis, which propose that neural responses to rhythm act as a core mechanism of selective attention that optimize processing at specific moments in time. Furthermore, they reveal that in addition to acting as a mechanism of early attentional selection, rhythm influences later, post-perceptual cognitive processes as events are transformed into memory.

## Introduction

Rhythmic temporal structures are common in our environment. Prior research has established that exposure to environmental rhythms influences perception and action by enhancing processing at specific moments of time that align with the rhythmic beat [1–4]. More recently, there has been growing interest in the influence of environmental rhythms on higher-order cognitive processes such as memory formation [5–8]. In a series of studies, Johndro and colleagues found that the timing of individual events within a rhythmic temporal stream influenced memory formation [9]. Specifically, stimuli presented in alignment with the timing of a background rhythm (on-beat) were better remembered in subsequent tests of memory than stimuli presented out-of-alignment (off-beat). This rhythmic modulation of memory (RMM) occurred even when rhythmic temporal cues were task-irrelevant and presented in a different modality (auditory) than the target stimuli (visual). This suggests that rather than simply dividing attention at encoding and negatively impacting subsequent memory [10, 11], background rhythmic cues can guide domain-general attentional resources to specific moments in time and enhance the processing of information that occurs in alignment with the beat. In this way, rhythm entrains attentional oscillations, as proposed in the Dynamic Attending Theory [4, 12–14], and dynamically influences what environmental information will be effectively encoded into long-term memory.

Electrophysiological evidence further supports the notion that dynamic fluctuations in attention underlie rhythmic modulations of memory encoding. It is well established that neural activity synchronizes to the periodicity of external rhythms, evident in increased power and phase alignment of neural oscillations at the same frequency as the external rhythm [1, 15, 16]. The entrainment of low-frequency neural oscillations, particularly in the delta range, has been shown to modulate cortical excitability dynamically over time, such that high excitability states align with the beat of the rhythm [14, 17]. This rhythmic shift in neural excitability has been proposed to act as a core mechanism of selective attention that optimizes stimulus processing at specific moments in time (in phase with the rhythmic beat) [17–19]. Support for this Oscillation Selection Hypothesis comes from numerous studies demonstrating enhanced perceptual processing of stimuli which appear at rhythmically-predicted moments in time [1, 17, 18, 20–24]. Recent work by Hickey and colleagues (2020) suggests that neural entrainment to low-frequency rhythm also modulates higher-order cognitive processing and the encoding of events into long-term memory [25]. In that study, participants incidentally encoded a series of visual objects which occurred in the context of background, rhythmic music with a steady beat [25]. Like the prior behavioral study by Johndro and colleagues [9], objects either appeared in-synchrony or out-of-synchrony with the background beat. Afterwards, participants completed a subsequent memory test (in silence). Results revealed increased electrophysiological power and inter-trial phase coherence at the perceived beat frequency (1.25 Hz) during encoding, indicating that neural activity tracked the rhythm of the musical beat. Importantly, enhanced neural tracking during encoding was associated with superior subsequent memory for in-synchrony compared to out-of-synchrony objects during a later memory test. While these results indicate that neural responses to rhythm influence memory formation at specific moments in time, an important outstanding question is the specific mechanism by which external rhythms influence stimulus processing en route to memory formation.

One possibility is that rhythmic temporal cues influence memory encoding by modulating early sensory or perceptual processing of stimuli. According to the Oscillation Selection Hypothesis, neural entrainment to external rhythms acts as an early sensory gain control mechanism that amplifies stimulus-driven neural activity at specific moments in time (i.e., in phase with the rhythm) [2, 17, 18]. Indeed, selective attention is known to modulate the amplitude of early stimulus-evoked potentials associated with initial sensory/perceptual processing [26–28]. For example, a well-established finding in the spatial attention literature is that directing attention to the location where a stimulus will appear enhances the amplitude of visual ERP components associated with initial stimulus processing, such as the N1 [26, 29]. These early amplitude modulations typically occur over occipital electrode sites and have been interpreted as reflecting gain control or amplification mechanisms within early visual pathways that enhance stimulus perception [26, 27]. In a recent study, Escoffier and colleagues extended these results in the temporal domain and found that the presentation of visual stimuli at temporally-predicted moments (in synchrony with a background auditory beat) enhances the amplitude of the N1 component over posterior electrode sites [30]. By this view, rhythm could influence visual memory encoding by enhancing early perceptual processing of stimuli that appear in synchrony with the beat.

In addition to influencing early sensory-perceptual processing, rhythmic temporal cues could also influence memory encoding by modulating later, post-perceptual stages of stimulus processing. It is well known that attention can bias stimulus processing at multiple stages of information processing [26–28, 31, 32]. Prior studies have shown later ERP components associated with post-perceptual stimulus identification and evaluation (e.g., N2, P3) [33–37] are sensitive to temporal orienting cues that direct attention to particular moments in time [32, 38, 39]. These later effects of temporal attention are typically greatest over more central electrode sites (e.g., Cz, Fz) compared to earlier effects of visual attention [32, 38]. It has also been suggested that manipulations of temporal attention may have their greatest effect on these later, post-perceptual stages of information processing [32, 36, 40]. In support of this proposal, effects of temporal orienting on early visual ERP components are not always observed and have been shown to depend on the perceptual demands of the task and the nature of the temporal orienting cues [22, 32, 40, 41–44]. Thus, the effect of rhythm on memory encoding could primarily reflect changes in later, post-perceptual stages of information processing.

Findings from the memory literature provide additional support for the possibility that the effect of rhythm on memory may reflect changes in later, post-perceptual stages of stimulus processing. Electrophysiological studies have identified a more sustained frontal slow wave (FSW) occurring later in time (>500ms after stimulus presentation) that predicts whether or not a stimulus will be later remembered or forgotten (subsequent memory) [45–47]. This ERP subsequent memory effect is most commonly observed over frontal electrodes in task contexts that strengthen memory encoding (e.g., deep vs. shallow processing, semantic vs. non-semantic classification) and has been linked to more elaborative, frontally-mediated cognitive processing that leads to the formation of a more durable memory trace [47–54]. Although there has been little research into the effects of temporal orienting on electrophysiological responses occurring during this later time window, modulations of this more sustained frontal response may be particularly relevant to effective memory encoding.

To date, only one prior study has attempted to identify the locus of rhythmic effects on memory encoding using ERPs [8]. In this study, participants explicitly encoded visual targets which appeared rhythmically (i.e., within blocks of trials in which visual stimuli were separated by a regular interstimulus interval) or arrhythmically (i.e., within blocks of trials in which visual stimuli were separated by an irregular interstimulus interval). The timing of stimulus presentation had a significant effect on memory encoding, with participants demonstrating superior subsequent memory for stimuli presented in the rhythmic compared to the arrhythmic context. Rhythmic presentation also influenced stimulus-evoked neural responses at encoding, modulating both the early visual N1 component occurring ~200ms after stimulus presentation, as well as later post-perceptual amplitudes starting ~400ms after stimulus presentation (post-perceptual N2/P3 components were not investigated). These results suggest that the presentation of stimuli within a global rhythmic temporal structure can influence both early perceptual as well as later cognitive stages of stimulus processing. However, modulation of both early and later ERP components was negatively associated with the effect of rhythm on memory performance, raising questions about the behavioral relevance of these evoked neural responses to rhythm. In addition, it is important to note that this study was unable to test whether evoked responses differ according to the timing of stimulus presentation within a rhythmic temporal stream (e.g., on-beat vs. off-beat). Such a comparison is critical in order to test the hypothesis that rhythmic temporal cues influence memory encoding by guiding attention to expected moments in time.

The current study leveraged the high temporal resolution of ERPs to explore how the timing of stimulus presentation *within* a rhythmic temporal structure influences different stages of stimulus processing during memory encoding. During EEG recording, participants incidentally encoded visual stimuli that appeared either in synchrony (on-beat) or out-of-synchrony (off-beat) with a background, auditory rhythm. Previously, Hickey and colleagues (2020) demonstrated that superior memory for visual stimuli that appear on-beat compared to off-beat at encoding was related to fidelity by which participants’ neural activity tracked the beat (oscillatory entrainment) [25]. The current study built upon these findings to examine the mechanism by which rhythm influences memory performance. Specifically, we investigated how rhythm influences stimulus-evoked neural responses (ERPs) during memory encoding in order to determine whether the effect of rhythm on stimulus processing occurs earlier or later in the processing stream. We first examined whether the timing of visual stimulus presentation within a background, auditory rhythm (on-beat vs. off-beat) influenced attention-related ERPs during early (perceptual) stages of processing and/or later (post-perceptual) stages of processing. If the rhythmic modulation of memory reflects attentional selection early in the processing stream (e.g., perceptual selection), the amplitudes of early perceptual ERPs (e.g., N1) should differ for on-beat and off-beat stimuli. If the rhythmic modulation of memory reflects attentional selection after perceptual processing is complete (e.g., post-perceptual selection) or changes in higher-order stimulus processing (e.g., cognitive control), later ERPs (N2, P3, FSW) should differ for on-beat and off-beat stimuli. In addition, motivated by prior observations of substantial individual variability in the magnitude of rhythmic effects on neural processing and behavior [7, 9, 23, 25, 43, 55–58], we performed an additional analysis to investigate whether individual differences in the mnemonic effects of rhythm were related to the magnitude of ERP responses to rhythm.

## Materials and methods

### Participants

Our sample consisted of 36 participants (24 female, 12 male) between the ages of 18–31 years (*M =* 23, *SD =* 3.32) who were recruited from Tufts University and the surrounding community. Participants were either compensated $15/hour or received course credit for participation. All participants had normal or corrected-to-normal eyesight and hearing, were fluent English speakers, and were right-handed. Additionally, participants were screened for history of neurological illness, brain injury, substance use, and psychiatric diagnosis. The current research was approved by the Tufts University Social, Behavioral, and Educational Research Institutional Review Board. All participants provided written informed consent prior to participation.

### Procedure

**Fig 1:**
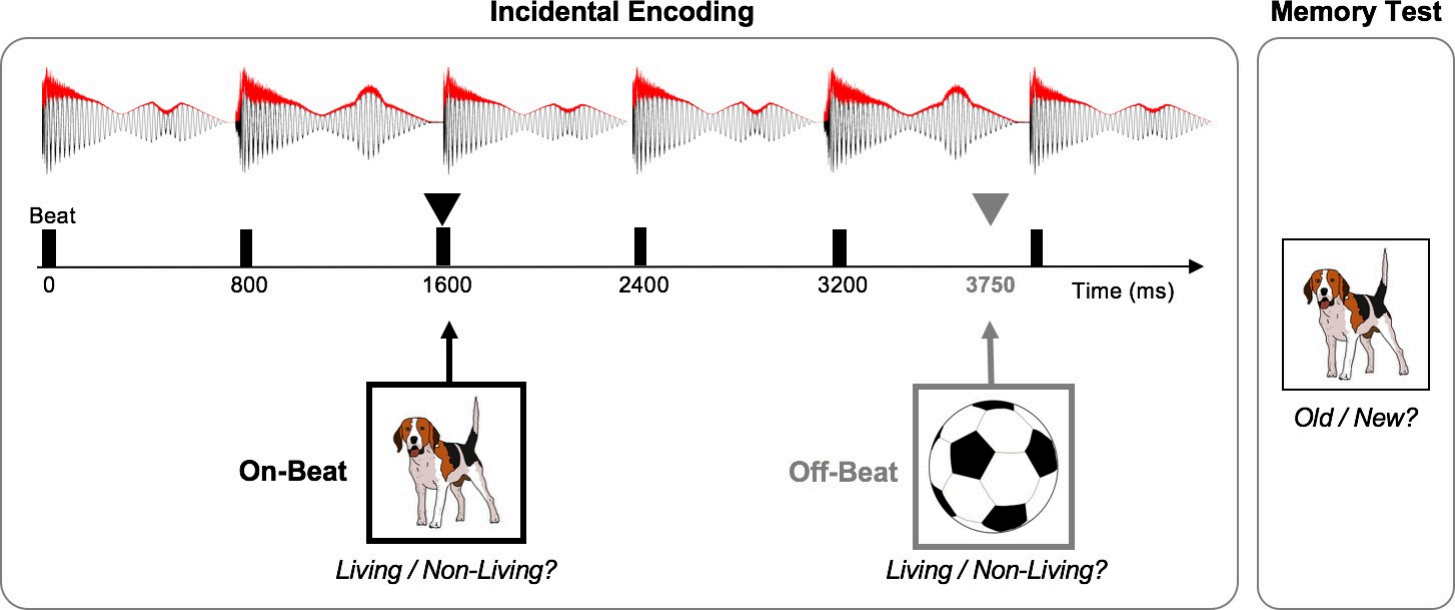
Behavioral paradigm. Participants viewed full color or black and white pictures of objects during incidental encoding while listening to background, rhythmic music with a steady beat. Each picture was presented for 750ms and participants made a semantic decision (living / non-living) about the object depicted in the picture. Pictures appeared either in-synchrony (on-beat) with the background rhythm or out-of-synchrony (off-beat) with the background rhythm (250ms prior to the beat). Following this incidental encoding task, participants performed a surprise recognition memory test (in silence) in which they decided whether presented pictures of objects had been previously shown during the encoding block (old) or had not been previously shown (new). An example of the musical waveform is shown at the top of the figure in black with its amplitude envelope depicted in red. Vertical black lines indicate the timing of the beat (800ms ISI, 1.25 Hz). Example images are displayed in the bottom portion of the figure with arrows indicating the time of image onset.

This study is a new analysis of the EEG data collected as part of the experiment previously reported by Hickey and colleagues [25]. Participants performed an incidental encoding task in which they were presented 120 images from the Multilingual Picture Database (MultiPic) [59]. Of the 120 images, 60 images depicted animate objects and 60 images depicted inanimate objects. During encoding, participants passively listened to background, instrumental music that did not contain words or lyrics (Fig 1). The background music was created in Garage Band and was designed to contain a steady beat with a tempo of 75bpm or 1.25 Hz (inter-beat interval = 800ms) and a 4/4 metrical structure. Each image was presented in the center of the screen for 750ms and participants were instructed to make a semantic classification as quickly and accurately as possible about whether the image was living (animate) or non-living (inanimate). The timing between images was jittered (*M* = 6.4s, *SD* = 1.25s) with interstimulus intervals ranging from 3.75s to 7.75s. Critically, images were presented either in-synchrony with the background rhythm (on-beat) or 250ms prior to the beat (off-beat). The order of the images was semi-randomized to ensure that in each third of the experiment an equal number of animate/inanimate and on/off beat images were presented and no more than six images of the same condition (on/off) appeared consecutively. EEG was recorded continuously throughout the incidental encoding period, which lasted approximately 13 minutes.

After the incidental encoding period, participants were immediately given a self-paced surprise memory test which occurred in silence. During the memory test, all 120 images from the encoding period and 60 new (lure) images were presented one at a time and participants decided whether they had previously seen the image in the first part of the experiment by making a yes/no button press on a keyboard. After making their memory decision, participants rated how confident they were in their decision (low or high). EEG was not recorded during the memory test.

### EEG recording and preprocessing

As described in detail previously [25], EEG was recorded using a BioSemi Active-Two amplifier system (Biosemi, Amsterdam, Netherlands) from 32 Ag/AgCl scalp electrodes and two reference electrodes place on the left and right mastoids. Two additional electrodes were also placed around the eyes to monitor eye movements. Since the BioSemi system uses active electrodes in which the amplifier is located within the electrodes, it was not necessary to check electrode impedance prior to recording. EEG pre-processing was completed using EEGlab and custom MATLAB scripts. All EEG signals recorded with a sampling rate of 1024 Hz and subsequently down-sampled to 512 Hz. Scalp electrode recordings were referenced to the average of the two mastoid electrodes and filtered using a 0.1 Hz high pass and 50 Hz low pass filter. Artifacts in the signal consistent with eye-blinks and muscle movements were removed using independent components analysis. Finally, the continuous signal was epoched from -2000ms to 2000ms surrounding visual stimulus presentation. Each epoch only contained one visual stimulus and epochs containing artifacts were manually rejected, yielding an average of 54.89 (*SD* = 4.46) on-beat epochs and 55.03 (*SD* = 4.18) off-beat epochs. Epochs were then baselined from -100ms to 0ms. Event related potentials were separately generated for on-beat and off-beat stimuli by averaging trials for each condition within each participant. Average event related potentials were also generated for the comparison of individuals who demonstrated greater memory for on-beat compared to off-beat stimuli (rhythmic modulation of memory; RMM group) and individuals who did not (no-RMM group).

### EEG data analysis

EEG analysis focused on the early N1 component associated with perceptual processing, as well as later ERP components associated with post-perceptual processing (N2, P3) and subsequent memory (frontal slow wave; FSW). For the N1, analysis time windows and occipital electrodes of interest were selected based on prior studies investigating rhythmic effects on perception [8, 30, 36]. Specifically, the peak of the N1 was determined based on the average waveform over bilateral posterior electrodes (O1, O2) where the N1 is typically largest and mean amplitudes were calculated and analyzed across a 20ms latency window around the peak (197-217ms). Slightly larger analysis time windows were selected for the later components following the approach used in prior studies investigating temporal cuing effects on stimulus processing [32, 39] and subsequent memory [8, 46]. Mean amplitudes for the N2 were calculated across a 40ms window around its peak (281-321ms) [32] and mean amplitudes for the P3 were calculated across a 100ms window around its peak (350-450ms) [39]. For these later N2/P3 attentional components, analysis focused on more frontocentral electrodes (Cz, Fz) given the topography of post-perceptual attentional effects previously observed in the literature [32, 36, 46, 50]. Mean amplitudes of the frontal slow wave were calculated across a cluster of frontal electrodes (Fz, FP1, FP2) during a time window ranging from 450-750ms based on the timing and topography of prior subsequent memory effects observed in the literature [47–54].

### Behavioral data analysis

Memory performance was measured by calculating the proportion of correctly identified old images (hits) and the proportion of incorrectly identified new images (false alarms) separately for on-beat and off-beat stimuli to calculate a measure of d-prime (d’) for each condition (on-beat, off-beat). Analysis of memory performance was collapsed over confidence ratings to increase power and to replicate prior work [9, 25]. Trials that were inaccurately responded to during encoding or trials with a reaction time >2SD from the mean during encoding were excluded from the analysis. Rhythmic modulation of memory (RMM) index scores were generated by subtracting the d’ measure for off-beat trials from the d’ for on-beat trials. A summary of the behavioral results is provided in Table 1.

**Table 1:** Behavioral performance.

		Reaction Time at Encoding (s)		Accuracy at Encoding (%)		Memory Performance (d')	
		*M*	*SD*	*M*	*SD*	*M*	*SD*
**Total**							
	On-Beat	0.64	0.09	96.90	4.56	1.53	0.59
	Off-Beat	0.65	0.09	96.96	3.19	1.48	0.59
	Difference	0.01	0.01			0.05	0.23
**RMM**							
	On-Beat	0.65	0.11	96.16	5.57	1.58	0.65
	Off-Beat	0.66	0.11	96.48	3.67	1.38	0.62
	Difference	0.01	0.01			0.19	0.12
**No-RMM**							
	On-Beat	0.63	0.05	98.08	1.85	1.45	0.47
	Off-Beat	0.63	0.05	97.73	2.14	1.62	0.53
	Difference	0.003	0.02			-0.18	0.16

Behavioral performance during memory encoding and at test across all participants, participants who showed rhythmic modulation of memory (RMM; n = 22), and individuals who did not show rhythmic modulation of memory (No-RMM; n = 14). Difference scores in reaction time at encoding are calculated by taking the difference between off and on (Off–On), while difference scores for memory performance are calculated by taking the difference between on and off (On–Off). M = Mean, SD = Standard Deviation.

### Statistical analysis

Separate statistical analyses were performed for each component/time window of interest. We first examined the effect of stimulus timing within the rhythmic temporal stream by comparing mean amplitudes for on-beat and off-beat trials. For the early N1, mean amplitudes were entered into a repeated-measures ANOVA with within-subject factors of timing (on-beat, off-beat) and electrode site (O1, O2). For the later attentional components (N2, P3), mean amplitudes were entered into separate repeated-measures ANOVAs with within-subject factors of timing (on-beat, off-beat) and electrode site (Cz, Fz). For the frontal slow wave associated with subsequent memory (FSW), mean amplitudes were entered into a repeated-measures ANOVA with within-subject factors of timing (on-beat, off-beat) and electrode site (Fz, FP1, FP2). We also conducted additional exploratory analyses to investigate the relationship between neural responses to rhythm and individual variability in the effect of rhythm on memory performance. First, we investigated whether amplitude modulations differed in participants who demonstrated a rhythmic modulation of memory effect (RMM group; n = 22) and those who did not demonstrate a rhythmic modulation of memory effect (No-RMM group; n = 14). Though exploratory and limited by the relatively small sample size in each group, this analysis was motivated by the prior observation of significant individual differences in the effect of rhythm on neural activity and memory performance [7, 8, 25, 58], as well as the finding that significant effects of rhythm on memory encoding only occur in individuals demonstrating strong neural responses to rhythm [25]. Next, we conducted Pearson correlations and sequential multiple regressions to further explore the relationship between stimulus-evoked ERP responses and the effect of rhythm on behavioral performance (rhythmic modulation of memory index) across individuals. All analyses were completed using SPSS version 25 and the PROCESS version 3.5 macro.

## Results

### Behavioral results

Behavioral performance is reported in Table 1. As described previously [25], reaction times during encoding were significantly faster for stimuli that were presented in-synchrony with the background beat compared to stimuli that were presented out-of-synchrony with the background beat (*Z = -*2.25, *p* = .01, one-tailed, *r* = .37), consistent with the proposal that rhythmic temporal cues orient attention to particular moments in time. During the subsequent memory test, memory performance was numerically greater for on-beat stimuli (*M* = 1.53, *SD* = .59) compared to off-beat stimuli (*M* = 1.48, *SD* = 0.59) across participants. However, there was considerable individual variability in the degree to which rhythm influenced subsequent memory performance, where some individuals showed the predicted effect (on > off), while others showed the reverse effect (off > on) (RMM index range = -.54 to .49, *SD* = .23, c.f. Fig 3C of [25]). As a result, there was no statistically significant difference between on-beat and off-beat memory across participants (*t*(35) = 1.28, *p* = .21, one-tailed, *d* = .21). Previously, Hickey and colleagues (2020) found that the magnitude of the effect of rhythm on memory performance across individuals is related to the strength of participants’ neural responses to rhythm (neural entrainment), and that greater memory for stimuli presented on-beat versus off-beat at encoding (rhythmic modulation of memory) is only observed in participants who demonstrate strong neural entrainment at the beat frequency [25]. This motivated separate exploratory analysis of individuals demonstrating a rhythmic modulation of memory effect (RMM; n = 22) and individuals who did not demonstrate a rhythmic modulation of memory effect (No-RMM; n = 14) in the present study. Behavioral data split by group is also presented in Table 1. In the RMM group, on-beat images were responded to faster than off-beat images at encoding (*Z* = -2.29, *p* = .02, *r* = .49) and were also better remembered than off-beat images in the subsequent memory test (*t*(21) = -2.63, *p* = .016, *d* = .56). In contrast, the no-RMM group did not demonstrate a significant difference in RTs for on-beat versus off-beat images at encoding (*Z* = -.79, *p* = .43, *r* = .21) and subsequent memory was reduced in the No-RMM group for on-beat compared to off-beat trials (*t*(13) = -4.02, *p* = .001, *d* = .15). There was no significant difference in response bias (c) between the RMM group (*M =* .08, *SD =* .36) and No-RMM group (*M =* .04, *SD =* .43).

### EEG results

#### Effects of stimulus timing

**Fig 2:**
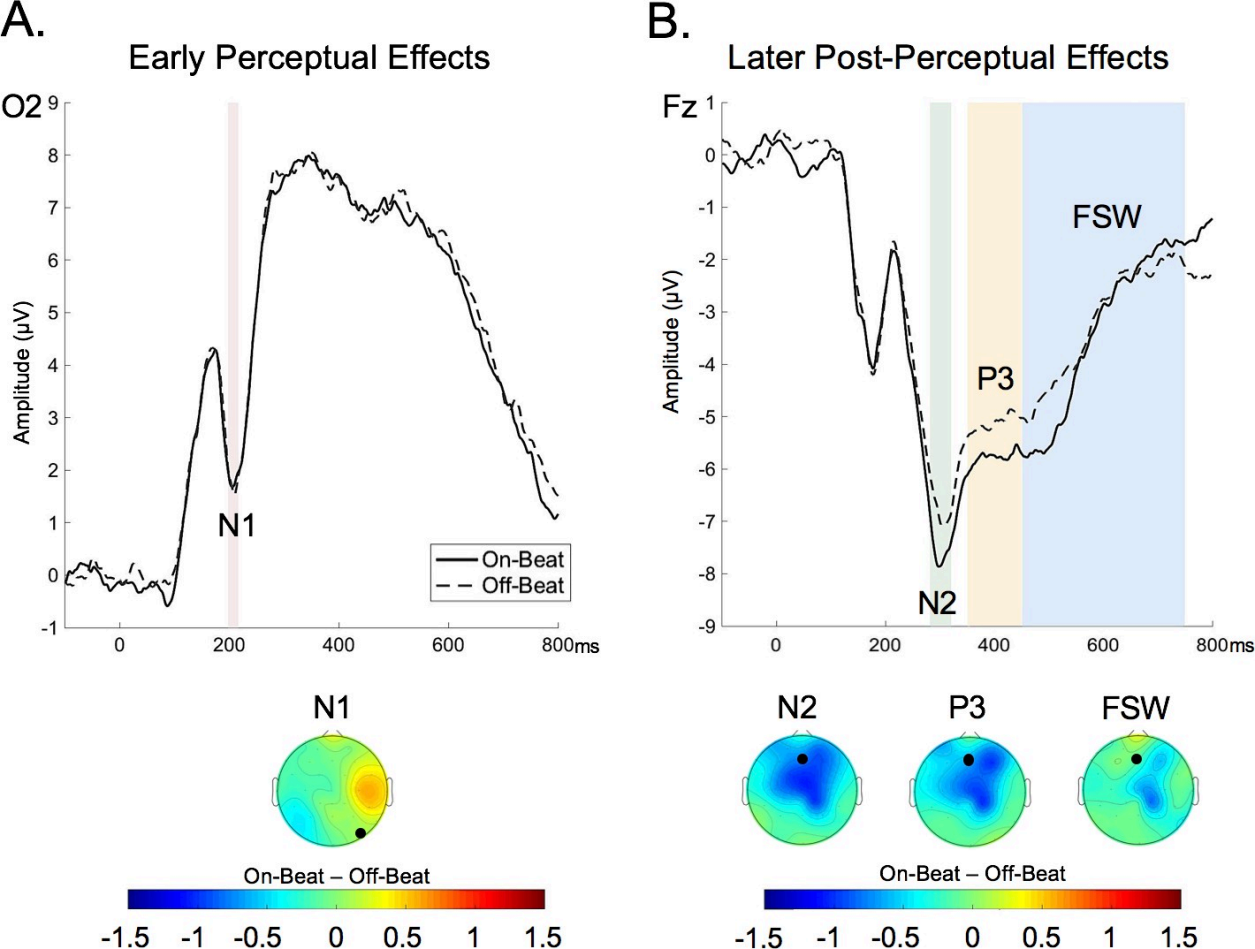
ERP waveforms for stimuli that appeared on-beat or off-beat during memory encoding. (A) Early perceptual effects are depicted at representative electrode O2. (B) Later post-perceptual effects are depicted at representative electrode Fz. The top of each panel depicts the ERP amplitudes for stimuli appearing in synchrony with the background beat (on-beat) or out-of-synchrony with the background beat (off-beat). On-beat ERPs are presented as solid black lines and off-beat ERPs are presented as hatched black lines. The bottom of each panel depicts a topographical plot of the difference between on-beat and off-beat trials across the scalp, with the representative electrode depicted in the ERP labeled as a black dot.

We first investigated whether the timing of visual stimulus presentation with respect to the background, auditory beat (on-beat vs. off-beat) influenced the amplitude of early perceptual (N1) or later post-perceptual (N2, P3, FSW) ERPs (Fig 2). Stimulus timing did not affect the amplitude of the early N1 (Fig 2A), with no main effect of stimulus timing, electrode, nor timing x electrode interaction (*p*s > .14). In contrast, the timing of stimulus presentation did affect the amplitude of the later N2 and the P3 components (Fig 2B). For the N2, there was a main effect of stimulus timing (*F*(1,35) = 9.39, *p* = .004, η^2^_p_ = .21), reflecting more negative amplitudes for on-beat compared to off-beat stimuli. There was also a main effect of electrode (*F*(1,35) = 12.84, *p* = .001, η^2^_p_ = .27), but no timing x electrode interaction (*F* < 1). For the P3 component, the main effect of stimulus timing approached significance (*F*(1,35) = 3.80, *p* = .06, η^2^_p_ = .10), with more positive amplitudes for off-beat compared to on-beat stimuli. There was also a main effect of electrode (*F*(1,35) = 34.69, *p* < .001, η^2^_p_ = .50), but no timing x electrode interaction (*F* < 1). In contrast to the effects of stimulus timing on the N2 and P3, the timing of stimulus presentation did not influence the amplitude of the FSW (*F* < 1). There was also not a main effect of electrode (*F*(1,35) = 1.37, *p* = .26, η^2^_p_ = .04), nor timing x electrode interaction (*F* < 1).

#### Rhythmic modulation of memory (RMM) effects

**Fig 3:**
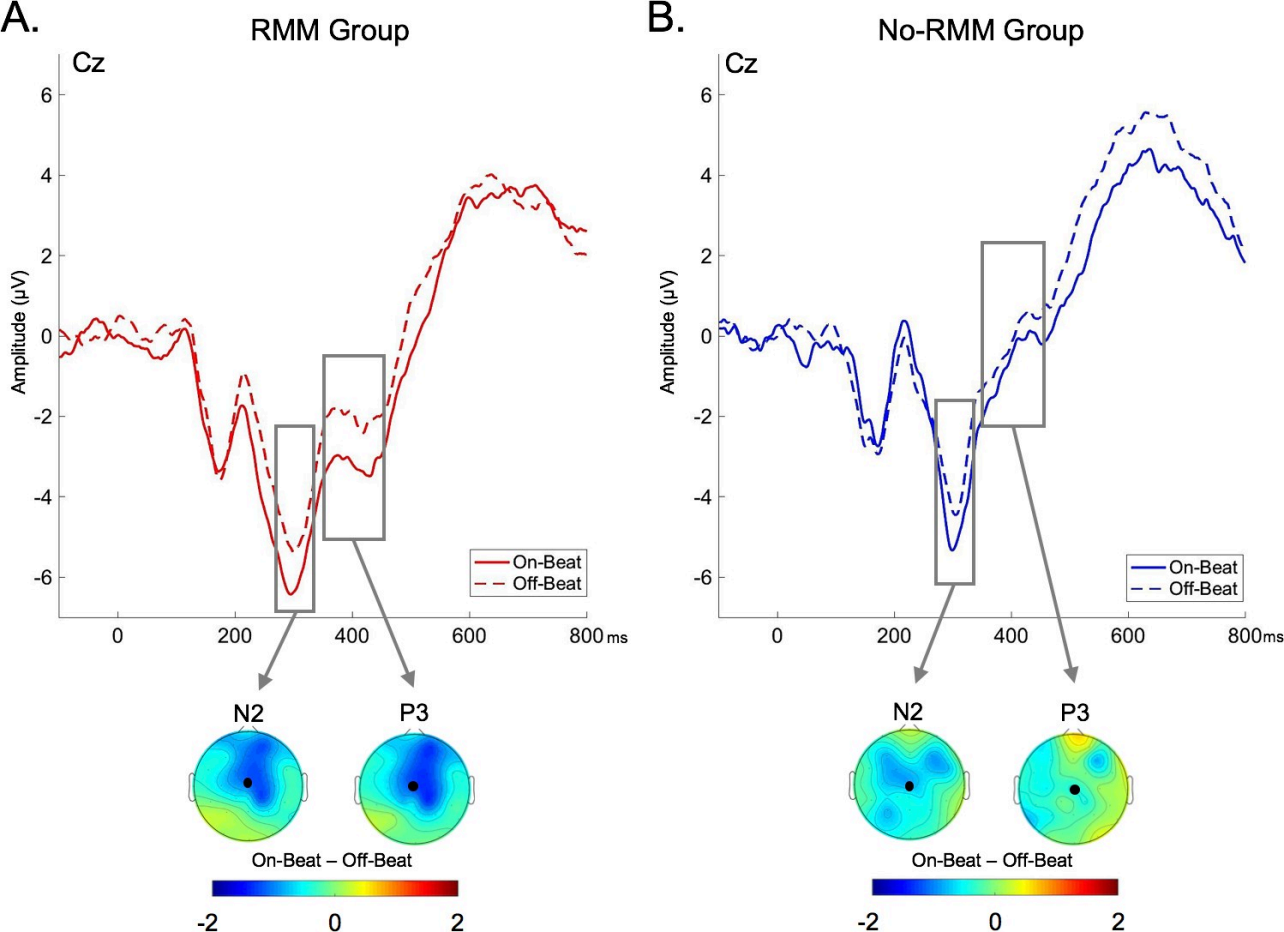
ERP waveforms for trials that either appeared on-beat or off-beat during memory encoding. (A) ERP waveforms in participants who demonstrated a subsequent memory benefit for on-beat compared to off-beat trials (Rhythmic Modulation of Memory; RMM). (B) ERP waveforms in participants who did not demonstrated a subsequent memory benefit for on-beat compared to off-beat trials (No Rhythmic Modulation of Memory; No-RMM). The top of each panel depicts the ERP amplitudes for stimuli appearing in synchrony with the background beat (on-beat) or out-of-synchrony with the background beat (off-beat). On-beat ERPs are presented as solid lines and off-beat ERPs are presented as hatched lines. The bottom of each panel depicts a topographical plot of the difference between on-beat and off-beat trials across the scalp, with the representative electrode depicted in the ERP labeled as a black dot.

The above results reveal that background auditory rhythms influence post-perceptual processing of to-be-remembered information as indexed by the N2 and P3 ERPs. We next performed two exploratory analyses which leveraged the variability present in individuals’ mnemonic response to rhythm in order to more closely investigate how these ERP effects might be related to memory performance. First, we compared the amplitudes of ERP components of interest in participants who did or did not demonstrate a positive effect of rhythm on subsequent memory (RMM, No-RMM). Amplitudes were entered into a repeated-measures ANOVA with within-subjects factors of timing (on-beat, off-beat) and electrode (Cz, Fz), and between-subjects factors of group (RMM, No-RMM). For both the N2 and P3 components, no timing x group interaction was present in the overall ANOVA (N2: *F*(1,34) = .71, *p* = .41, η^2^_p_ = .02; P3: *F*(1,34) = 1.40, *p* = .24, η^2^_p_ = .04). However, effects of stimulus timing were numerically greater in the RMM than the No-RMM groups for both the N2 and the P3. Based on prior evidence that rhythmic modulation of memory effects are greater in participants who demonstrate stronger neural responses to rhythm [7, 25, 58], we next conducted a follow-up analysis comparing on-beat to off-beat trials in each group separately (Fig 3). For the N2, ERP amplitudes were enhanced for on-beat compared off-beat trials in participants who demonstrated a rhythmic modulation of memory effect (RMM group; *F*(1,21) = 9.52, *p* = .006, η^2^_p_ = .31). In contrast, there was not a significant difference between on-beat and off-beat amplitudes in participants who did not demonstrate a rhythmic modulation of memory effect (No-RMM group; *F*(1,13) = 1.32, *p* = .27, η^2^_p_ = .09). A similar pattern was present for the P3 component: ERP amplitudes were enhanced for on-beat compared off-beat trials in participants who demonstrated a rhythmic modulation of memory effect (RMM group; *F*(1,21) = 5.78, *p* = .03, η^2^_p_ = .22) while ERP amplitudes for on-beat and off-beat trials did not differ in participants who did not demonstrate a rhythmic modulation of memory effect (No-RMM group; *F*(1,13) = .08, *p* = .79, η^2^_p_ = .006).

**Fig 4:**
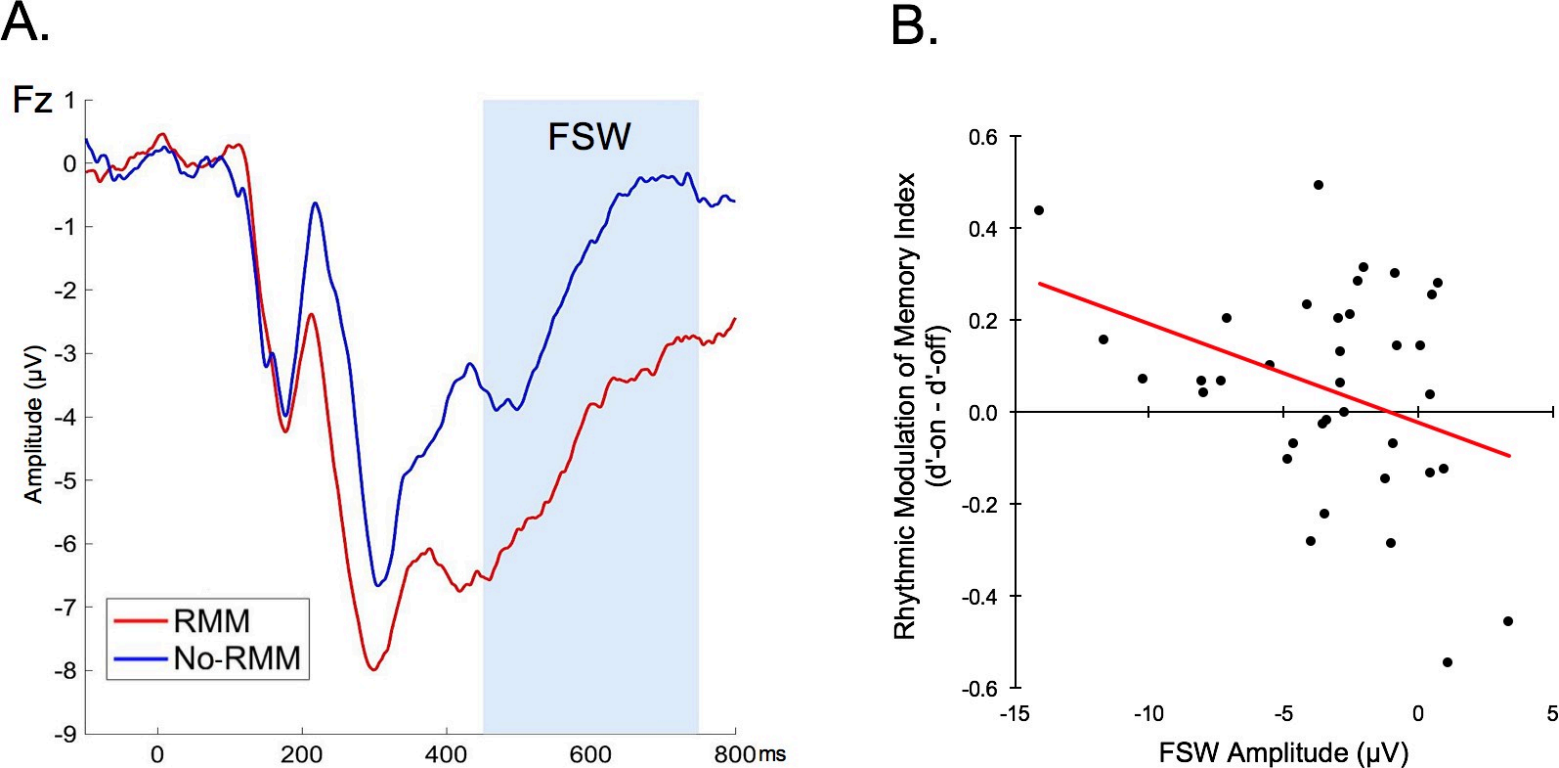
Rhythmic modulation of memory effect. (A) The amplitude of the frontal slow wave (FSW) ERP was reduced in participants who demonstrated a subsequent memory benefit for on-beat compared to off-beat trials (Rhythmic Modulation of Memory; RMM) compared to those who did not (No-RMM) (ERP waveforms for each group are depicted at representative electrode Fz). (B) The amplitude of the frontal slow wave (FSW) negatively correlated with the rhythmic modulation of memory index (difference in memory for on-beat versus off-beat trials) across participants. Error bars = SEM. * p < .05.

We next investigated whether stimulus-evoked responses in any of the components of interest (N1, N2, P3, FSW) differed for the RMM and No-RMM groups regardless of stimulus timing. Mean amplitudes for each component were entered into separate mixed ANOVAs with within-subjects factors of electrode and between-subjects factors of group (RMM, No-RMM). For the early N1 component, there was no main effect of group (RMM, No-RMM) nor group x electrode interaction (*Fs* < 1). This was also the case for the N2 and P3 components. In contrast, there was a significant main effect of group for the FSW (*F*(1,34) = 4.78, *p* = .04 η^2^_p_ = .12) (Fig 4A), with reduced amplitudes in the RMM group compared to the No-RMM group.

#### Brain-behavior correlations

Following the approach used in prior studies [8, 25], we next investigated whether there was a significant correlation between the magnitude of stimulus-evoked neural responses and the magnitude of the effect of rhythm on subsequent memory across participants. In order to minimize the number of exploratory correlations, we focused on representative electrodes that showed a significant difference between timing (Cz: N2, P3) or between RMM grouping (Fz: FSW). First, we examined the post-perceptual N2 and P3 components which showed a significant effect of stimulus timing. There was no correlation between the rhythmic modulation of memory index (d’-on minus d’-off) and amplitude differences (on-beat minus off-beat trials) across individuals for the N2 or the P3 (*p*s > .20). Next, we examined the later frontal positivity which showed a significant effect of group (RMM, No-RMM). There was a significant negative correlation between the rhythmic modulation of memory index and the FSW amplitude across individuals (*r*(36) = -.36, *p* = .03) (Fig 4C).

**Fig 5:**
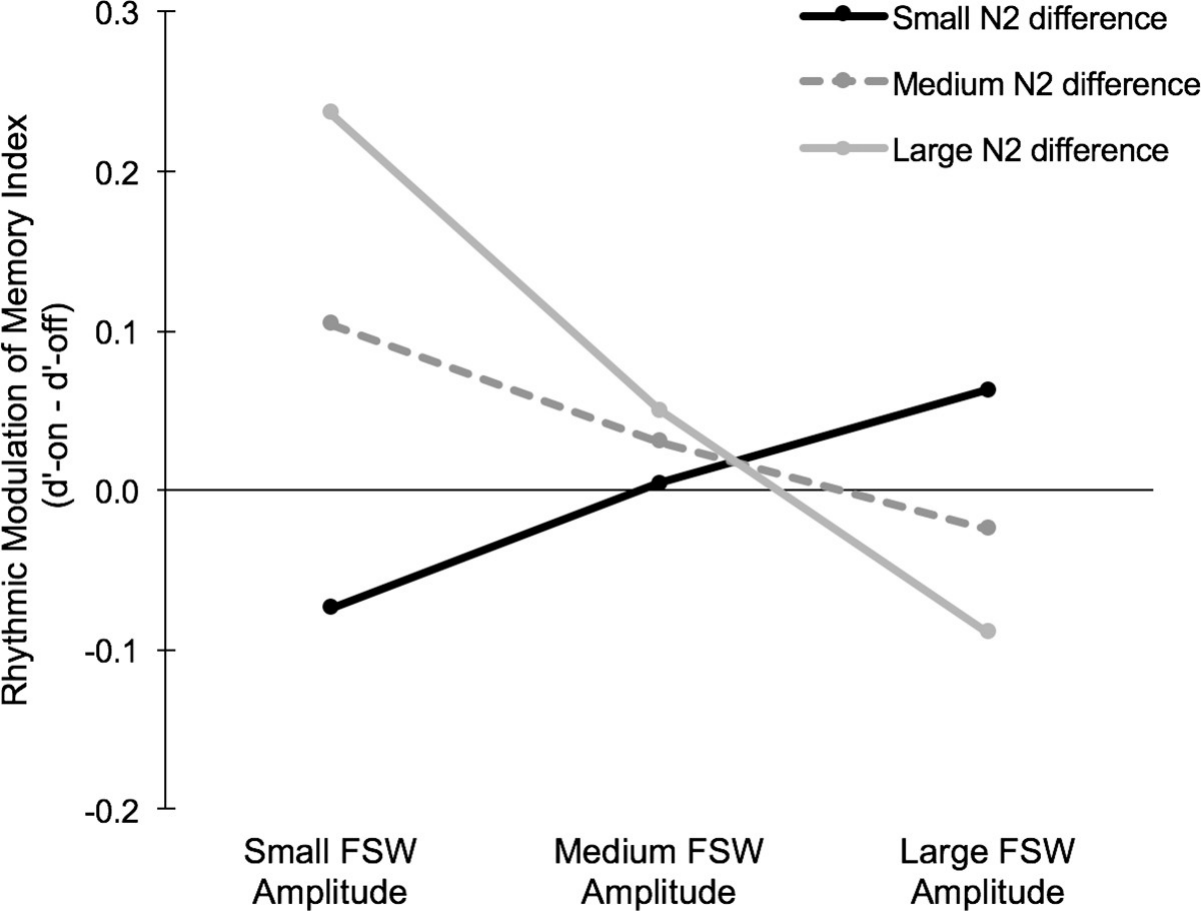
Relationship between post-perceptual ERPs and effects of rhythm on subsequent memory. The relationship between frontal slow wave (FSW) amplitudes and the rhythmic modulation of memory (better memory for on-beat versus off-beat trials) is moderated by individual differences in participants’ neural response to rhythm earlier in the processing stream (N2). Given that FSW amplitudes were significantly different for the RMM and no RMM group at electrode Fz, the FSW amplitudes in the regression analysis were measured at electrode Fz. Since differences in the N2 were seen at electrode Cz, the N2 effect (amplitude difference for on-beat–off-beat trials) is measured at electrode Cz. Small, medium, and large bins for the FSW and N2 effects were defined based on the SD of each predictor. Rhythmic modulation of memory index = d’-On–d’-Off.

We then conducted two sequential multiple regression analyses to explore whether individual differences in earlier neural responses to rhythm (N2/P3 modulations for on-beat vs. off-beat trials) interact with the later ERP effects (FSW) to predict behavioral performance. Predictors and outcome variables were all linearly related and followed a normal distribution. No multivariate outliers were present in either model and there were no extreme univariate outliers (>3SD from the mean) present in the outcome variable or any of the predictors. In the first model, the N2 effect (amplitude difference for on-beat vs. off-beat trials at electrode site Cz) and the FSW effect (mean amplitude at electrode site Fz) were entered as continuous predictors with the rhythmic modulation of memory index (subsequent memory for on-beat minus off-beat trials) as the dependent variable. Together, the N2 and FSW predictors did not account for a significant amount of variance in memory performance (*R*^*2*^ = .13; *F*(3,32) = 2.46, *p* = .10), although FSW amplitude still individually predicted RMM (*B* = -.02, *β* = -.35, *p* = .04). However, the addition of the interaction term in the second step significantly improved the model *(R*^*2*^ *change* = .11, *F*(1,32) = 4.45, *p* = .04) such that it accounted for a significant amount of variation in behavioral performance (*R*^*2*^ = .24; *F*(3,32) = 3.29, *p* = .03). Importantly, while neither the N2 (*B* = .03, *β* = .24, *p* = .26) nor the FSW (*B* < .001, *β* = .002, *p* = .99) were reliable predictors of the rhythmic modulation of memory in this model, the interaction term was significant (*B* = .02, *β* = .58, *p* = .04). This reveals that individual differences in participants’ neural response to rhythm earlier in the processing stream moderates the relationship between the FSW amplitude and the rhythmic modulation of memory. Specifically, individuals who had the greatest N2 difference for on-beat and off-beat trials (e.g., greatest attentional orienting effect) and the lowest FSW amplitudes (e.g., potentially an index of reduced higher-level cognitive processing) demonstrated the largest effect of rhythm on subsequent memory performance (Fig 5). The second multiple regression analysis mirrored the first analysis, with the only difference being that the P3 effect was entered as a continuous predictor rather than the N2 effect. This model did not account for a significant amount of variance in memory performance regardless of whether or not the interaction term was included (*p*s > .09), suggesting that the P3 effect did not moderate the relationship between the FSW and the rhythmic modulation of memory. Post analysis diagnostics of both regression models confirmed the reliability of our results, as there were no influential cases present (Cook’s Distance < 1), the assumption of homoscedasticity was met, and multicollinearity between predictors was not present (Tolerance > .10; VIF < 10).

## Discussion

The current study provides novel electrophysiological evidence that rhythmic, environmental cues influence long-term memory formation by guiding attention to specific moments in time. During incidental encoding, stimulus-evoked neural responses differed for visual images that appeared in synchrony versus out-of-synchrony with a background auditory beat. Amplitude differences were present in post-perceptual ERP components (e.g., N2, P3) associated with temporal attention and were greatest in participants demonstrating superior subsequent memory for on-beat compared to off-beat trials (rhythmic modulation of memory). The behavioral effect of rhythm on memory performance was also related to the amplitude of a later frontal slow wave during the time window of classic subsequent memory effects. Together, these results support the proposal put forth in the Dynamic Attending Theory that rhythm directs domain-general attentional resources to specific moments in time [4, 14], and suggests that the dynamic effect of rhythm on memory encoding reflects post-perceptual attentional selection as well as downstream changes in higher-order cognitive processing.

A key finding of the current study was that the timing of visual stimulus presentation during encoding (on-beat vs. off-beat) influenced the amplitude of later ERP components (e.g., N2, P3) rather than earlier ERP components (e.g., N1). This finding is consistent with prior studies using non-rhythmic temporal cues to direct attention in time, which have also observed effects of temporal attention on visually-evoked N2 and P3 components [32, 38]. Importantly, the current results extend this prior work by demonstrating that background, rhythmic cues that occur in a different modality (auditory) than the target stimuli (visual) also modulate post-perceptual ERPs. While other prior work has shown that rhythmic cues can also influence earlier N1 components associated with initial stimulus processing [8, 30], the modulation of sensory/perceptual ERPs is not always observed following manipulations of temporal attention and may depend on the nature of the temporal cues or task context [22, 40, 41–44]. For example, in prior studies demonstrating rhythmic effects on the N1, participants performed perceptually-demanding target detection/discrimination tasks [8, 30]. Correa and colleagues have argued that temporal attention consistently modulates later ERPs (e.g., N2, P3), but that early ERPs (e.g., N1) are only affected by temporal orienting cues in the context of tasks that place high demands on perceptual processing (e.g., target discrimination) [32]. In the current study, perceptual demands were relatively low, as participants were tasked with making a semantic decision about each stimulus as it appeared. Thus, it is possible that earlier effects could emerge in a more perceptually-demanding task. Importantly, our finding that rhythmic temporal cues influence stimulus processing at relatively late stages of processing suggests that in addition to acting as a mechanism of early attentional selection [1, 2, 17, 18, 60, 61], rhythmic temporal cues can also act as a mechanism of selection at post-perceptual stages of processing.

In prior studies, effects of temporal attention on the N2 and P3 components have been interpreted as reflecting attention-related modulations of decision-related or response-related processing [32, 36, 38, 39]. While the direction of the amplitude modulation of the N2 and P3 for temporally-predicted versus temporally-unpredicted stimuli varies across studies, amplitude modulations like those observed in the current study (increased N2 amplitudes and decreased P3 amplitudes) have been observed when stimuli appear at attended locations/times or are contextually expected (i.e., not deviant) [37, 62–66]. In the current study, amplitude modulations of the N2 and P3 could therefore reflect the tuning or sharpening of decision-related or response-related processes which in turn optimize the encoding of temporally-predicted stimuli that appear in synchrony with the beat (e.g., serve as a temporal filter) [67, 68]. Support for this proposal comes from the finding that in addition to being better remembered, on-beat stimuli in the current study were also more rapidly classified during incidental encoding compared to off-beat stimuli. Furthermore, post-hoc analysis indicates that while the amplitude of the N2/P3 did not correlate with overall response times during encoding, there was a significant correlation between P3 amplitude (at electrode Cz) and the response facilitation for on-beat versus off-beat stimuli at encoding (*r*(36) = -.34, *p* = .04), whereby participants demonstrating faster classification times for on-beat versus off-beat stimuli during encoding also demonstrated greater P3 amplitude reductions. Though the functional significance of the N2/P3 amplitude modulations represent an important area for future research, these results are congruent with the proposal that rhythmic modulation of mid-latency components reflects facilitated decision or response-related processing.

A more direct link between rhythmic effects on post-perceptual stimulus processing and memory encoding comes from the finding that the N2 and P3 amplitudes not only differed according to the timing of stimulus presentation within the rhythmic stream, but also differed according to whether or not participants demonstrated an effect of stimulus timing on subsequent memory. Specifically, a significant difference in N2 and P3 amplitudes for on-beat versus off-beat stimuli was only present in participants who demonstrated a positive rhythmic modulation of memory effect (better subsequent memory for on-beat versus off-beat stimuli). Though this pattern of results should be interpreted with caution given the small sample size of each participant subgroup, it converges with prior evidence suggesting a relationship between neural and behavioral responses to rhythm [23, 25, 43, 55–58]. Furthermore, this result suggests that the way in which temporally-predicted (on-beat) versus temporally-unpredicted (off-beat) stimuli are initially processed impacts how well they are later remembered. However, this effect of rhythm on post-perceptual ERPs was not directly associated with the effect of rhythm on later memory performance, as neither the N2 nor P3 modulations correlated with the mnemonic benefit for on-beat versus off-beat stimuli. Instead, the rhythmic effects on attention-related components (specifically the N2) interacted with amplitude modulations of the later frontal slow wave to predict memory performance. This raises the intriguing possibility that the effect of rhythm on memory encoding reflects an interaction between earlier attentional selection and downstream cognitive processing.

In contrast to the N2 and P3 ERP effects, the timing of stimulus presentation (on-beat vs. off-beat) within the rhythmic stream did not influence the amplitude of the later frontal slow wave. Instead, the amplitude of the frontal slow wave differed according to whether or not participants demonstrated better subsequent memory for on-beat vs. off-beat stimuli, with reduced amplitudes in participants demonstrating greater rhythmic modulation of memory. The directionality of this amplitude difference may initially seem at odds with the typical finding in the subsequent memory literature of greater amplitudes for items that are later remembered versus forgotten. However, the direction of the ERP subsequent memory effect can differ according to the nature of the to-be-remembered stimulus (e.g., words vs. non-words) [47]. Additionally, it is important to note that in the current study modulations of the frontal slow wave do not reflect subsequent memory performance per se (i.e., whether individual stimuli will be later remembered or forgotten) but rather reflect individual differences in the *effect of rhythm* on subsequent memory performance. Thus, rather than serving as an index of general encoding success, modulations of the frontal slow wave may reflect the degree to which higher-level cognitive operations occurring later in the processing stream are required for stimulus processing. Indeed, prior studies have found that the frontal slow wave is modulated by higher-level control demands [69–71]. In the context of the current study, demands on controlled processing may be reduced when environmental rhythms can be leveraged to direct attention in time. For example, greater frontal slow wave amplitudes in the current study could reflect more intensive or prolonged semantic processing in participants who are less able to leverage rhythmic temporal cues to enhance memory encoding. This interpretation aligns with the finding that individual differences in earlier ERP effects of temporal orienting (e.g., N2 amplitude difference for on-beat versus off-beat trials) moderated the relationship between the amplitude of the frontal slow wave and the rhythmic modulation of memory. Additional support for this interpretation comes from the observation that the amplitude of the frontal slow wave negatively correlated with the rhythmic modulation of memory index across participants. Interestingly, Jones and Ward recently observed a similar result, whereby later ERP modulations were negatively related to the effects of rhythm on memory performance across participants [8]. Although future studies should further investigate the specific processes indexed by these late ERP effects, together these results suggest that later stages of information processing play an important role in the effects of rhythm on memory encoding.

While the results of the current study support the hypothesis that external rhythms dynamically modulate memory encoding by influencing post-perceptual stimulus processing, there are several limitations and outstanding questions that should be addressed in future research. One limitation is the relatively small size of the sample, especially when considering the comparison of individuals in the RMM and No-RMM groups. The separation of participants into these groups was motivated by the large degree of variability observed in participants’ behavioral response to rhythm. Even within the No-RMM group, some participants demonstrated no effect of rhythm on memory performance (i.e., no difference between memory for on-beat and off-beat stimuli) and others even showed the opposite effect as might be predicted (i.e., better memory for off-beat compared to on-beat stimuli). While this opposite effect may seem unexpected, it is consistent with what has been previously observed in the literature, where some individuals demonstrate a behavioral benefit due to rhythm and others demonstrate a behavioral cost [7, 9, 58]. Importantly, such individual variability has been linked to the fidelity by which participants’ neural activity tracks the beat of the rhythm [7, 23, 25, 56, 58]. In this way, individuals whose neural activity more precisely synchronizes to the periodicity of the beat generate more accurate temporal predictions (and demonstrate superior memory for on-beat stimuli), whereas individuals whose neural activity less precisely synchronizes to the periodicity of the beat generate less accurate (faster or slower) temporal predictions (and may demonstrate potentially superior memory for off-beat stimuli). Motivated by this work, we were interested in exploring whether a similar relationship between mnemonic and neural responses to rhythm (as measured by stimulus-evoked ERPs) was present in our data. Our finding that post-perceptual ERPs differ according to whether or not participants demonstrate a mnemonic benefit of rhythm converges with this prior work and highlights the importance of considering individual variability in neural and behavioral responses to rhythm. Thus, while the results of our individual differences analysis should be considered exploratory and treated with caution given their low statistical power, these results add to accumulating evidence in the literature that variability in individuals’ neural responses to rhythm play an important role in the effect of rhythm on behavior [7, 9, 23, 25, 43, 55–58]. An important aim of future research will be to replicate the finding of a relationship between rhythmic modulation of memory and post-perceptual evoked responses in larger groups of participants.

Future work should also investigate the important outstanding question of whether rhythm also impacts memory by influencing neural activity *before* the onset of a stimulus. Prior research has demonstrated that pre-stimulus neural activity can predict whether or not visual events are effectively encoded into memory and have been interpreted as reflecting the degree to which encoding-related attentional processes are prepared ahead of stimulus presentation [72, 73]. Temporal orienting of attention has also been shown to influence anticipatory neural responses before the onset of a stimulus [39, 74]. Thus, external rhythms that direct attention to particular moments in time may also influence memory encoding by regulating cortical excitability prior to the onset of a stimulus. To address this possibility, future work should assess how pre-stimulus activity, such as the pre-stimulus phase of entrained oscillations or pre-stimulus alpha/beta power, influences the rhythmic modulation of memory.

A second outstanding question is whether there is a relationship between the evoked neural responses observed in the current study and previously reported effects of rhythm on oscillatory entrainment. Using the same paradigm, Hickey and colleagues recently demonstrated that rhythmic effects on memory encoding are closely related to how strongly rhythm is represented in the brain (i.e., oscillatory entrainment to the rhythmic beat) [25]. Although it is important to note that the ERPs in the current study were measured in response to the onset of the to-be-remembered visual stimuli rather than the onset of background auditory beat, and are therefore unlikely to be measuring the same type of neural response captured by entrainment measures, these two types of neural responses could be related and together may serve to better predict the effect of rhythm on memory encoding [19, 75–77]. Future research should aim to clarify how these different types of neural responses to rhythm have independent and/or interactive effects on memory encoding [64, 76].

## References

[1] Calderone DJ, Lakatos P, Butler PD, Castellanos FX. Entrainment of neural oscillations as a modifiable substrate of attention. Trends Cogn Sci. 2014;18(6):300–9. 10.1016/j.tics.2014.02.005 24630166 PMC4037370

[2] Haegens S, Zion Golumbic E. Rhythmic facilitation of sensory processing: A critical review. Neurosci Biobehav Rev. 2018;86:150–65. 10.1016/j.neubiorev.2017.12.002 29223770

[3] Nobre AC, van Ede F. Anticipated moments: Temporal structure in attention. Nat Rev Neurosci. 2018;19(1):34–48. 10.1038/nrn.2017.141 29213134

[4] Jones MR. Time will tell: A theory of dynamic attending. Oxford: Oxford University Press; 2019.

[5] Thavabalasingam S, O'Neil EB, Zeng Z, Lee AC. Recognition memory is improved by a structured temporal framework during encoding. Front Psychol. 2015;6:2062 10.3389/fpsyg.2015.02062 26834673 PMC4720003

[6] Clouter A, Shapiro KL, Hanslmayr S. Theta phase synchronization is the glue that binds human associative memory. Curr Biol. 2017;27(20):3143–8.e6. 10.1016/j.cub.2017.09.001 28988860

[7] Wang D, Clouter A, Chen Q, Shapiro KL, Hanslmayr S. Single-trial phase entrainment of theta oscillations in sensory regions predicts human associative memory performance. J Neurosci. 2018;38(28):6299–309. 10.1523/JNEUROSCI.0349-18.2018 29899027 PMC6596103

[8] Jones A, Ward EV. Rhythmic temporal structure at encoding enhances recognition memory. J Cogn Neurosci. 2019;31(10):1549–62. 10.1162/jocn_a_01431 31172861

[9] Johndro H, Jacobs L, Patel AD, Race E. Temporal predictions provided by musical rhythm influence visual memory encoding. Acta Psychol (Amst). 2019;200:102923 10.1016/j.actpsy.2019.102923 31759191

[10] Craik F, Govoni R, Naveh-Benjamin M, Anderson ND. The effects of divided attention on encoding and retrieval processes in human memory. J Exp Psychol. 1996;125(2):159–80. 10.1037//0096-3445.125.2.159 8683192

[11] Naveh- Benjamin M, Guez J, Marom M. The effects of divided attention at encoding on item and associative memory. Mem Cogn. 2003;31(7):1021–35. 10.3758/BF03196123 14704017

[12] Jones MR. Time, our lost dimension: Toward a new theory of perception, attention, and memory. Psychol Rev. 1976;83(5):323–55. 10.1037/0033-295X.83.5.323 794904

[13] Jones MR, Boltz M. Dynamic attending and responses to time. Psychol Rev. 1989;96(3):459–91. 10.1037/0033-295x.96.3.459 2756068

[14] Large EW, Jones MR. The dynamics of attending: How people track time-varying events. Psychol Rev. 1999;106(1):40 10.1037/0033-295X.106.1.119

[15] Thut G, Miniussi C, Gross J. The functional importance of rhythmic activity in the brain. Curr Biol. 2012;22(16):R658–63. 10.1016/j.cub.2012.06.061 22917517

[16] Nozaradan S. Exploring how musical rhythm entrains brain activity with electroencephalogram frequency-tagging. Philos Trans R Soc Lond B Biol Sci. 2014;369(1658):20130393 10.1098/rstb.2013.0393 25385771 PMC4240960

[17] Schroeder CE, Lakatos P. Low-frequency neuronal oscillations as instruments of sensory selection. Trends Neurosci. 2009;32(1):9–18. 10.1016/j.tins.2008.09.012 19012975 PMC2990947

[18] Lakatos P, Karmos G, Mehta AD, Ulbert I, Schroeder CE. Entrainment of neuronal oscillations as a mechanism of attentional selection. Science. 2008;320(5872):110–3. 10.1126/science.1154735 18388295

[19] Lakatos P, Gross J, Thut G. A new unifying account of the roles of neuronal entrainment. Curr Biol. 2019;29(18):R890–R905. 10.1016/j.cub.2019.07.075 31550478 PMC6769420

[20] Mathewson KE, Prudhomme C, Fabiani M, Beck DM, Lleras A, Gratton G. Making waves in the stream of consciousness: entraining oscillations in EEG alpha and fluctuations in visual awareness with rhythmic visual stimulation. J Cogn Neurosci. 2012;24(12):2321–33. 10.1162/jocn_a_00288 22905825

[21] Henry MJ, Herrmann B, Obleser J. Entrained neural oscillations in multiple frequency bands comodulate behavior. Proc Natl Acad Sci U S A. 2014;111(41):14935–40. 10.1073/pnas.1408741111 25267634 PMC4205645

[22] Tierney A, Kraus N. Neural entrainment to the rhythmic structure of music. J Cogn Neurosci. 2015;27(2):400–8. 10.1162/jocn_a_00704 25170794

[23] Doelling KB, Poeppel D. Cortical entrainment to music and its modulation by expertise. Proc Natl Acad Sci U S A. 2015;112(45):E6233–42. 10.1073/pnas.1508431112 26504238 PMC4653203

[24] Frey JN, Ruhnau P, Weisz N. Not so different after all: The same oscillatory processes support different types of attention. Brain Res. 2015;1626:183–97. 10.1016/j.brainres.2015.02.017 25721788

[25] Hickey P, Merseal H, Patel AD, Race E. Memory in time: Neural tracking of low-frequency rhythm dynamically modulates memory formation. Neuroimage. 2020;213:116693 10.1016/j.neuroimage.2020.116693 32135262

[26] Hillyard SA, Anllo-Vento L. Event-related brain potentials in the study of visual selective attention. Proc Natl Acad Sci U S A. 1998;95(3):781–7. 10.1073/pnas.95.3.781 9448241 PMC33798

[27] Hopfinger JB LS, Hillyard SA. The Cognitive Neurosciences: MIT Press; 2004

[28] Hillyard SA, Störmer VS, Feng W, Martinez A, McDonald JJ. Cross-modal orienting of visual attention. Neuropsychologia. 2016;83:170–8. 10.1016/j.neuropsychologia.2015.06.003 26072092

[29] Störmer VS, McDonald JJ, Hillyard SA. Cross-modal cueing of attention alters appearance and early cortical processing of visual stimuli. Proc Natl Acad Sci U S A. 2009;106(52):22456–61. 10.1073/pnas.0907573106 20007778 PMC2799760

[30] Escoffier N, Herrmann CS, Schirmer A. Auditory rhythms entrain visual processes in the human brain: evidence from evoked oscillations and event-related potentials. NeuroImage. 2015;111:267–76. 10.1016/j.neuroimage.2015.02.024 25701698

[31] Luck SJ, Woodman GF, Vogel EK. Event-related potential studies of attention. Trends Cogn Sci. 2000;4(11):432–40. 10.1016/s1364-6613(00)01545-x 11058821

[32] Correa A, Lupiáñez J, Madrid E, Tudela P. Temporal attention enhances early visual processing: A review and new evidence from event-related potentials. Brain Res. 2006;1076(1):116–28. 10.1016/j.brainres.2005.11.074 16516173

[33] Sutton S, Braren M, Zubin J, John ER. Evoked-potential correlates of stimulus uncertainty. Science. 1965;150(3700):1187–8. 10.1126/science.150.3700.1187 5852977

[34] Kutas M, McCarthy G, Donchin E. Augmenting mental chronometry: the P300 as a measure of stimulus evaluation time. Science. 1977;197(4305):792–5. 10.1126/science.887923 887923

[35] Luck SJ, Hillyard SA. Electrophysiological correlates of feature analysis during visual search. Psychophysiology. 1994;31(3):291–308. 10.1111/j.1469-8986.1994.tb02218.x 8008793

[36] Doherty JR, Rao A, Mesulam MM, Nobre AC. Synergistic effect of combined temporal and spatial expectations on visual attention. J Neurosci. 2005;25(36):8259–66. 10.1523/JNEUROSCI.1821-05.2005 16148233 PMC6725546

[37] Rolke B, Festl F, Seibold VC. Toward the influence of temporal attention on the selection of targets in a visual search task: An ERP study. Psychophysiology. 2016;53(11):1690–701. 10.1111/psyp.12734 27479494

[38] Miniussi C, Wilding EL, Coull JT, Nobre AC. Orienting attention in time. Modulation of brain potentials. Brain. 1999;122 (Pt 8):1507–18. 10.1093/brain/122.8.1507 10430834

[39] Rohenkohl G, Nobre AC. α oscillations related to anticipatory attention follow temporal expectations. J Neurosci. 2011;31(40):14076–84. 10.1523/JNEUROSCI.3387-11.2011 21976492 PMC4235253

[40] Griffin IC, Miniussi C, Nobre AC. Multiple mechanisms of selective attention: Differential modulation of stimulus processing by attention to space or time. Neuropsychologia. 2002;40(13):2325–40. 10.1016/s0028-3932(02)00087-8 12417462

[41] Correa A, Lupiáñez J, Tudela P. Attentional preparation based on temporal expectancy modulates processing at the perceptual level. Psychon Bull Rev. 2005;12(2):328–34. 10.3758/bf03196380 16082814

[42] Lange K. The ups and downs of temporal orienting: A review of auditory temporal orienting studies and a model associating the heterogeneous findings on the auditory N1 with opposite effects of attention and prediction. Front Hum Neurosci. 2013;7:263 10.3389/fnhum.2013.00263 23781186 PMC3678089

[43] Tierney A, Kraus N. Neural responses to sounds presented on and off the beat of ecologically valid music. Front Syst Neurosci. 2013;7:14 10.3389/fnsys.2013.00014 23717268 PMC3650712

[44] Bouwer FL, Honing H, Slagter HA. Beat-based and memory-based temporal expectations in rhythm: similar perceptual effects, different underlying mechanisms. J Cogn Neurosci. 2020:1–21. 10.1162/jocn_a_01529 31933432

[45] Paller KA, Kutas M, Mayes AR. Neural correlates of encoding in an incidental learning paradigm. Electroencephalogr Clin Neurophysiol. 1987;67(4):360–71. 10.1016/0013-4694(87)90124-6 2441971

[46] Paller KA, Wagner AD. Observing the transformation of experience into memory. Trends Cogn Sci. 2002;6(2):93–102. 10.1016/s1364-6613(00)01845-3 15866193

[47] Otten LJ, Sveen J, Quayle AH. Distinct patterns of neural activity during memory formation of nonwords versus words. J Cogn Neurosci. 2007;19(11):1776–89. 10.1162/jocn.2007.19.11.1776 17958481 PMC2443936

[48] Fabiani M, Karis D, Donchin E. Effects of mnemonic strategy manipulation in a von restorff paradigm. Electroencephalogr Clin Neurophysiol. 1990;75(1–2),22–35. 10.1016/0013-4694(90)90149-E 1688770

[49] Otten LJ, Henson RN, Rugg MD. Depth of processing effects on neural correlates of memory encoding: relationship between findings from across- and within-task comparisons. Brain. 2001;124(Pt 2):399–412. 10.1093/brain/124.2.399 11157567

[50] Guo C, Zhu Y, Ding J, Fan S, Paller KA. An electrophysiological investigation of memory encoding, depth of processing, and word frequency in humans. Neurosci Lett. 2004;356(2):79–82. 10.1016/j.neulet.2003.09.049 14746868

[51] Burke JF, Long NM, Zaghloul KA, Sharan AD, Sperling MR, Kahana MJ. Human intracranial high-frequency activity maps episodic memory formation in space and time. Neuroimage. 2014;85 Pt 2:834–43. 10.1016/j.neuroimage.2013.06.067 23827329 PMC4289670

[52] Richter FR, Yeung N. ERP correlates of encoding success and encoding selectivity in attention switching. PLoS One. 2016;11(12):e0167396 10.1371/journal.pone.0167396 27907075 PMC5131936

[53] Kamp SM, Bader R, Mecklinger A. ERP subsequent memory effects differ between inter-item and unitization encoding tasks. Front Hum Neurosci. 2017;11 10.3389/fnhum.2017.00030 28194105 PMC5276848

[54] Höltje G, Lubahn B, Mecklinger A. The congruent, the incongruent, and the unexpected: Event-related potentials unveil the processes involved in schematic encoding. Neuropsychologia. 2019;131:285–93. 10.1016/j.neuropsychologia.2019.05.013 31112723

[55] Herrmann B, Henry MJ, Haegens S, Obleser J. Temporal expectations and neural amplitude fluctuations in auditory cortex interactively influence perception. Neuroimage. 2016;124:487–97. 10.1016/j.neuroimage.2015.09.019 26386347

[56] Nozaradan S, Peretz I, Keller PE. Individual differences in rhythmic cortical entrainment correlate with predictive behavior in sensorimotor synchronization. Sci Rep. 2016;6 10.1038/srep20612 26847160 PMC4742877

[57] Assaneo MF, Ripollés P, Orpella J, Lin WM, de Diego-Balaguer R, Poeppel D. Spontaneous synchronization to speech reveals neural mechanisms facilitating language learning. Nat Neurosci. 2019;22:627–32. 10.1038/s41593-019-0353-z 30833700 PMC6435400

[58] Köster M, Martens U, Gruber T. Memory entrainment by visually evoked theta-gamma coupling. Neuroimage. 2019;188:181–87 10.1016/j.neuroimage.2018.12.002 30529173

[59] Duñabeitia JA, Crepaldi D, Meyer AS, New B, Pliatsikas C, Smolka E, et al MultiPic: A standardized set of 750 drawings with norms for six European languages. Q J Exp Psychol (Hove). 2018;71(4):808–16. 10.1080/17470218.2017.1310261 28326995

[60] Lange K, Röder B. Orienting attention to points in time improves stimulus processing both within and across modalities. J Cogn Neurosci. 2006;18(5):715–29. 10.1162/jocn.2006.18.5.715 16768372

[61] Auksztulewicz R, Myers NE, Schnupp JW, Nobre AC. Rhythmic temporal expectation boosts neural activity by increasing neural gain. J Neurosci. 2019;39(49):9806–17. 10.1523/JNEUROSCI.0925-19.2019 31662425 PMC6891052

[62] Rimmele J, Jolsvai H, Sussman E. Auditory target detection is affected by implicit temporal and spatial expectations. J Cogn Neurosci. 2011;23(5):1136–47. 10.1162/jocn.2010.21437 20146603 PMC2894284

[63] Schwartze M, Rothermich K, Schmidt-Kassow M, Kotz SA. Temporal regularity effects on pre-attentive and attentive processing of deviance. Biol Psychol. 2011;87(1):146–51. 10.1016/j.biopsycho.2011.02.021 21382437

[64] Kashiwase Y, Matsumiya K, Kuriki I, Shioiri S. Temporal dynamics of visual attention measured with event-related potentials. PLoS One. 2013;8(8):e70922 10.1371/journal.pone.0070922 23976966 PMC3747140

[65] Honing H, Bouwer FL, Háden GP. Perceiving temporal regularity in music: The role of auditory event-related potentials (ERPs) in probing beat perception. Adv Exp Med Biol. 2014;829:305–23. 10.1007/978-1-4939-1782-2_16 25358717

[66] Król M, El-Deredy W. The clash of expectancies: Does the P300 amplitude reflect both passive and active expectations? Q J Exp Psychol (Hove). 2015;68(9):1723–34. 10.1080/17470218.2014.996166 25688470

[67] Nieuwenhuis S, Aston-Jones G, Cohen JD. Decision making, the P3, and the locus coeruleus-norepinephrine system. Psychol Bull. 2005;131(4):510–32. 10.1037/0033-2909.131.4.510 16060800

[68] Gold JI, Shadlen MN. The neural basis of decision making. Annu Rev Neurosci. 2007;30:535–74. 10.1146/annurev.neuro.29.051605.113038 17600525

[69] Friederici AD, Hahne A, Mecklinger A. Temporal structure of syntactic parsing: Early and late event-related brain potential effects. J Exp Psychol Learn Mem Cogn. 1996;22(5):1219–48. 10.1037//0278-7393.22.5.1219 8805821

[70] Daltrozzo J, Wioland N, Kotchoubey B. The N400 and late positive complex (LPC) effects reflect controlled rather than automatic mechanisms of sentence processing. Brain Sci. 2012;2(3):267–97. 10.3390/brainsci2030267 24961195 PMC4061799

[71] Kamp SM, Endemann R, Domes G, Mecklinger A. Effects of acute psychosocial stress on the neural correlates of episodic encoding: Item versus associative memory. Neurobiol Learn Mem. 2019;157:128–38. 10.1016/j.nlm.2018.12.006 30553022

[72] Otten LJ, Quayle AH, Akram S, Ditewig TA, Rugg MD. Brain activity before an event predicts later recollection. Nat Neurosci. 2006;9(4):489–91. 10.1038/nn1663 16501566

[73] Otten LJ, Quayle AH, Puvaneswaran B. Prestimulus subsequent memory effects for auditory and visual events. J Cogn Neurosci. 2010;22(6):1212–23. 10.1162/jocn.2009.21298 19583467 PMC3881065

[74] Driver J, Frith C. Shifting baselines in attention research. Nat Rev Neurosci. 2000;1(2):147–8. 10.1038/35039083 11252778

[75] van Ede F, Quinn AJ, Woolrich MW, Nobre AC. Neural oscillations: sustained rhythms or transient burst-events? Trends Neurosci. 2018;41(7):415–7. 10.1016/j.tins.2018.04.004 29739627 PMC6024376

[76] Doelling KB, Assaneo MF, Bevilacqua D, Pesaran B, Poeppel D. An oscillator model better predicts cortical entrainment to music. Proc Natl Acad Sci U S A. 2019;116(20):10113–21. 10.1073/pnas.1816414116 31019082 PMC6525506

[77] Helfrich RF, Breska A, Knight RT. Neural entrainment and network resonance in support of top-down guided attention. Curr Opin Psychol. 2019;29:82–9. 10.1016/j.copsyc.2018.12.016 30690228 PMC6606401

